# Decreased MYC-associated factor X (MAX) expression is a new potential biomarker for adverse prognosis in anaplastic large cell lymphoma

**DOI:** 10.1038/s41598-020-67500-w

**Published:** 2020-06-25

**Authors:** Takahisa Yamashita, Morihiro Higashi, Shuji Momose, Akiko Adachi, Toshiki Watanabe, Yuka Tanaka, Michihide Tokuhira, Masahiro Kizaki, Jun-ichi Tamaru

**Affiliations:** 1 Department of Pathology, Saitama Medical Center, Saitama Medical University, Kawagoe, Japan; 2 Department of Pathology, Saitama Red Cross Hospital, Saitama, Japan; 3 Medical Genome Sciences, Frontier Sciences, The University of Tokyo, Tokyo, Japan; 4 Department of Hematology, Saitama Medical Center, Saitama Medical University, Kawagoe, Japan

**Keywords:** Cancer, Tumour biomarkers

## Abstract

MYC-associated factor X (MAX) is a protein in the basic helix-loop-helix leucine zipper family, which is ubiquitously and constitutively expressed in various normal tissues and tumors. MAX protein mediates various cellular functions such as proliferation, differentiation, and apoptosis through the MYC-MAX protein complex. Recently, it has been reported that MYC regulates the proliferation of anaplastic large cell lymphoma. However, the expression and function of MAX in anaplastic large cell lymphoma remain to be elucidated. We herein investigated MAX expression in anaplastic large cell lymphoma (ALCL) and peripheral T-cell lymphoma, not otherwise specified (PTCL-NOS) and found 11 of 37 patients (30%) with ALCL lacked MAX expression, whereas 15 of 15 patients (100%) with PTCL-NOS expressed MAX protein. ALCL patients lacking MAX expression had a significantly inferior prognosis compared with patients having MAX expression. Moreover, patients without MAX expression significantly had histological non-common variants, which were mainly detected in aggressive ALCL cases. Immunohistochemical analysis showed that MAX expression was related to the expression of MYC and cytotoxic molecules. These findings demonstrate that lack of MAX expression is a potential poor prognostic biomarker in ALCL and a candidate marker for differential diagnosis of ALCL and PTCL-NOS.

## Introduction

Anaplastic large cell lymphoma (ALCL) is an aggressive mature T-cell lymphoma that usually expresses the lymphocyte activation marker CD30 and often lacks expression of T-cell antigens, such as CD3, CD5, and CD7^1^. Histological patterns of ALCL are recognized as the so-called common pattern (most frequent: 60–70%), lymphohistiocytic pattern, small cell pattern, and Hodgkin-like pattern. ALCLs are commonly classified into systemic ALK-positive ALCL, systemic ALK-negative ALCL, and primary cutaneous ALCL (cALCL). ALK-positive ALCL has the *ALK* gene rearrangement that generates the ALK fusion protein and the product of translocation partner gene. Genetic changes in ALK-negative ALCL have been recently clarified, such as *Dual Specificity Phosphatase 22* (*DUSP22)* or *TP63* rearrangement, which is exclusive to *ALK* rearrangement^2–4^. Approximately 30% and 8% of ALK-negative ALCL patients have *DUSP22* and *TP63* rearrangement, respectively, and ALCL patients with *TP63* rearrangement have a worse prognosis, while patients with *DUSP22* rearrangement generally have an intermediate or good prognosis^2,5,6^. ALCL cases without these rearrangements are still classified into a “basket waste category”, triple-negative type^2^. cALCL, which is usually only located in the skin, has the most favorable outcome among the these ALCL subtypes.

MYC-associated factor X (MAX) is a protein in the basic helix-loop-helix leucine zipper (bHLHLZ) family that can homodimerize or heterodimerize with other bHLHLZ proteins, in particular, oncoprotein MYC^7^. MYC-MAX heterodimer activates transcription of target genes by binding to the E-box DNA sequence (CACGTG), while MAX-MAX homodimer competitively inhibits this transcription of MYC-associated genes. MYC can regulate cell proliferation, differentiation, and apoptosis in cooperation with MAX, and this binding to MAX is known to be necessary for *MYC* transcriptional activities^7^. Recently, it has been reported that MYC regulates the proliferation of aggressive mature T-cell lymphomas, ALCL, and peripheral T-cell lymphoma, not otherwise specified (PTCL-NOS). Differential diagnosis of these diseases can be difficult because of their immunophenotypic similarities^8–12^. MAX expression and function in ALCL remain to be elucidated, although MAX expression is absent in some solid cancers such as small cell lung cancer and gastric intestinal stromal tumor^13,14^, and MAX is considered a tumor suppressor gene^7,15,16^. In this study, we evaluated MAX expression in ALCL patients and examined the impact of MAX expression as a prognostic marker of ALCL. We also determined whether MAX expression can be a candidate biomarker to differentiate between ALCL and PTCL-NOS.

## Results

### MAX expression is decreased in lymphoma cell lines

**Figure 1:**
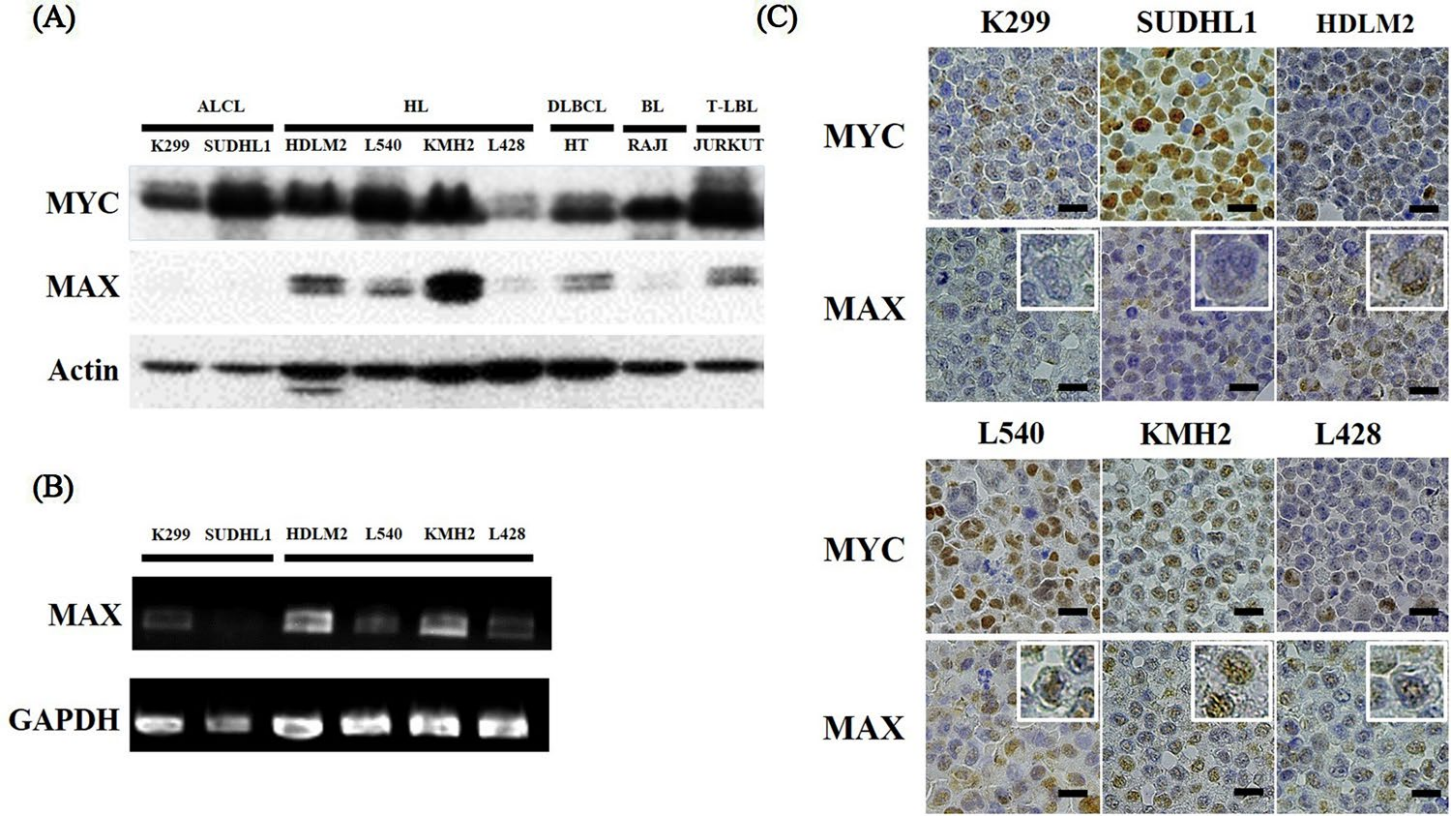
MAX expression in various lymphomas. (**A**) Western blotting, (**B**) reverse transcriptase PCR, and (**C**) immunohistochemical analysis of cell blocks from cell lines revealed that MAX expression was repressed in ALCL cell lines (inset with fourfold magnification). Bars: 50 μm. ALCL, anaplastic large cell lymphoma; HL, Hodgkin lymphoma; DLBCL, diffuse large B-cell lymphoma; BL, Burkitt lymphoma; T-LBL, T lymphoblastic lymphoma.

MYC and MAX expression in lymphoma-derived cell lines was assessed by western blotting. MAX expression was not observed in two ALCL cell lines, K299 and SUDHL1, whereas other cell types expressed MAX protein (Fig. 1A). MYC was expressed in all lymphoma cell lines analyzed. MAX mRNA expression was also decreased in K299 and SUDHL1 cells (Fig. 1B). Immunohistochemical analysis of cell blocks showed MYC expression in all cell lines, whereas MAX expression was at low level in two ALCL cell lines (Fig. 1C). From these results, we hypothesized that MAX expression is decreased in ALCL.

### MYC and MAX expression in ALCL and PTCL-NOS patients

**Figure 2:**
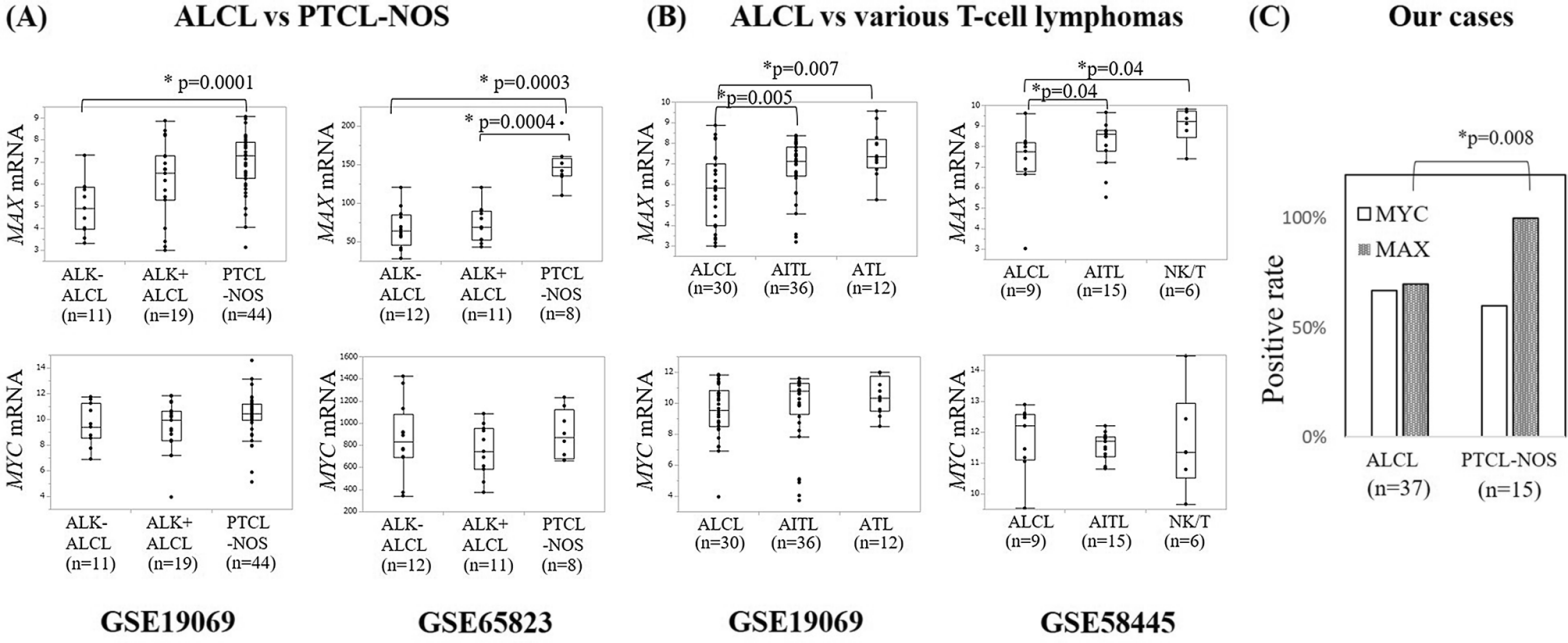
Comparison of *MAX* and *MYC* mRNA or protein expression between ALCL and the other T-cell lymphomas in other studies and our cases. (**A**) comparison between ALCL and PTCL-NOS in two public dataset (GSE19069 and GSE6823), (**B**) comparison between ALCL and the other major T-cell lymphomas in two public dataset (GSE19069 and GSE58445) and (**C**) comparison between ALCL and PTCL-NOS in our cases. *MAX* mRNA levels in ALCL, particularity ALK-negative ALCL, were lower than those in PTCL-NOS and were also lower than the other T-cell lymphomas, angioimmunoblastic T-cell lymphoma (AITL), adult T-cell lymphoma (ATL) and extranodal natural killer/T-cell lymphoma, nasal type (NK/T). However, *MYC* mRNA levels were similar between ALCL and the others. In our patients, the positive rate of MAX expression in ALCL was significantly lower than that in PTCL-NOS as well. ALK + ALCL, ALK-positive anaplastic large cell lymphoma; ALK-ALCL, ALK-negative anaplastic large cell lymphoma; PTCL-NOS, peripheral T-cell lymphoma, not otherwise specified.

To clarify whether MAX expression is at low level in ALCL, we analyzed two independent datasets from a public database (GSE19069 and GSE65823). *MAX* transcription in ALK-negative ALCL was lower than that in PTCL-NOS in both datasets. *MAX* expression in ALK-positive ALCL patients was significantly lower than in PTCL-NOS in the GSE65823 dataset, whereas *MYC* mRNA levels were comparable among these lymphomas (Fig. 2A). In the GSE 19069 dataset, *MAX* expression in ALK-positive ALCL patients was lower than in PTCL-NOS, though the difference was not statistically significant. This difference of *MAX* expression in ALK-positive ALCL between the datasets may be attributed to the percentage of tumor cells in each sample. Indeed, the *MAX* expression adjusted to *CD30* expression were comparable between ALK-positive and ALK-negative ALCL in both datasets (see Supplementary Fig. S1A, B online). Moreover, we investigated MAX protein expression by immunohistochemistry for 37 and 15 samples of pre-treatment ALCL and PTCL-NOS. Eleven of 37 ALCL patients (30%) lacked MAX expression, whereas 15 of 15 PTCL-NOS patients (100%) showed MAX expression (*p* = 0.008) (Fig. 2C, Supplementary Table S2 online). These results indicate that MAX may have a critical role in ALCL, and MAX expression may be a candidate biomarker to differentiate between ALCL and PTCL-NOS.

### MYC and MAX expression of various T-cell lymphomas

We also analyzed *MYC* and *MAX* mRNA expression levels in other T-cell lymphomas compared with ALCL. *MAX* mRNA expression in ALCL cases was significantly lower than that in other T-cell lymphomas (angioimmunoblastic T-cell lymphoma, adult T-cell leukemia/lymphoma, and extranodal natural killer/T-cell lymphoma, nasal type), regardless of *MYC* mRNA expression (Fig. 2B). Taken together, low MAX expression is specific for ALCL among T-cell lymphoma studied, regardless of MYC expression.

### Clinical characteristics according to MAX expression in ALCL patients

We compared clinical characteristics between ALCL patients with MAX expression (MAX-positive ALCL) and ALCL patients without MAX expression (MAX-negative ALCL). As shown in Table 1, there were no significant differences in clinical features between MAX-positive and MAX-negative ALCL patients, such as invasion site (*p* = 0.295 to 1.000), clinical stage (*p* = 0.940), IPI (*p* = 0.940), and serum lactate dehydrogenase (*p* = 0.908). Furthermore, no significant difference in MAX expression was observed in cALCL (*p* = 0.391). However, serum soluble interleukin-2 receptor level was higher in MAX-negative ALCL than in MAX-positive ALCL, although the difference was not statistically significant.

**Table 1:** Clinical characteristics of ALCL patients according to MAX expression.

Characteristics	MAX-positive ALCL	MAX-negative ALCL	*P* value
Total number of patients	26	11	–
Age (y), median age (range)	57 (11–78)	66 (19–81)	0.245
Sex (male/female)	14/12	7/4	0.773
Invasion site			
Lymphoid tissue	12/26 (46%)	8/11 (73%)	0.295
Skin	10/26 (31%)	1/11 (9%)	0.060
Lung	5/26 (19%)	3/11 (27%)	1.000
Bone marrow	2/26 (8%)	2/11 (18%)	0.580
Liver	2/26 (8%)	1/11 (9%)	1.000
Soft tissue	3/26 (12%)	0/11 (0%)	0.535
Gastrointestinal region	1/26 (4%)	1/11 (9%)	1.000
Kidney	1/26 (4%)	0/11 (0%)	1.000
Primary cutaneous ALCL	7/26 (27%)	1/11 (9%)	0.391
Stage			
I/II/III/IV/unknown	10/1/4/8/3	4/1/2/4/0	0.940
IPI			
L/L-I/I-H/H/unknown	10/8/1/4/3	4/4/1/2/0	0.940
LDH (IU/L) median (range)	244 (143–915)	239 (143–1,092)	0.908
sIL-2R (U/ml) median (range)	1,680 (240–38,500)	6,185 (1,010–90,000)	0.099
Chemotherapy	16/20 (80%)	8/11 (73%)	0.676

ALCL, anaplastic large cell lymphoma; LDH, lactate dehydrogenase; sIL-2R, soluble interleukin-2 receptor; IPI, International Prognostic Index; L, low risk; L-I, low-intermediate risk; I-H, intermediate-high risk; H, high risk.

### Clinical outcomes according to MAX expression in ALCL patients

**Figure 3:**
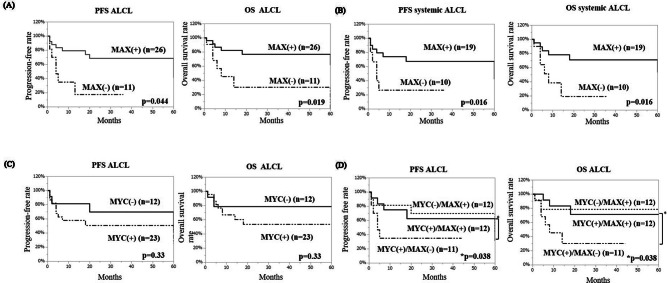
Clinical outcome of ALCL stratified by MAX and MYC expression. (**A**) ALCL patients (including cALCL) lacking MAX expression had poor PFS and OS. (**B**) Systemic ALCL patients (excluding cALCL) lacking MAX expression had a worse prognosis than patients with MAX expression, as in (**A**). (**C**) MYC-positive ALCL patients tended to have a worse prognosis than MYC-negative ALCL patients. (**D**) Both PFS and OS of MAX-negative patients were lower than those of MAX-positive patients, regardless of MYC expression. ALCL, anaplastic large cell lymphoma; cALCL, primary cutaneous anaplastic large cell lymphoma; OS, overall survival; PFS, progression-free survival.

We analyzed clinical outcomes of 37 ALCL patients. The 3-year progression-free survival (PFS) and overall survival (OS) rates of all ALCL patients were 55% and 64%, respectively. The 3-year PFS rate was significantly lower in MAX-negative ALCL than in MAX-positive ALCL (18% vs 64%, *p* = 0.044). The 5-year OS rate in MAX-negative ALCL (n = 11) was also significantly lower than that in MAX-positive ALCL (n = 26) (30% vs 77%, *p* = 0.019) (Fig. 3A). As cALCL is known to have a good prognosis and is usually classified as an independent entity from systemic ALCL, we analyzed PFS and OS again in only systemic ALCL patients (Fig. 3B). In these patients, 3-year PFS and OS rates in MAX-negative ALCL (n = 10) were significantly lower than those in MAX-positive ALCL (n = 19) (PFS, 23% vs 63%, *p* = 0.016; OS, 19% vs 66%, *p* = 0.016, respectively). Because MYC-MAX heterodimerization is essential for MYC-driven oncogenesis, we stratified patients according to MYC expression and conducted Cox analysis. In our cohort, there were no cases of MYC(-) and MAX(-). MYC-positive ALCL had a poorer prognosis than MYC-negative ALCL, although the difference was not statistically significant (Fig. 3C). Of note, MAX-positive ALCL had a better prognosis than MAX-negative ALCL, regardless of MYC expression (Fig. 3D).

To evaluate the possibility that MAX expression serves as an independent prognostic factor in ALCL, we conducted univariate and multivariate Cox regression analyses for PFS and OS using the following variables: sex, IPI, MYC expression, MAX expression, and ALK expression. In univariate and multivariate analyses for PFS, significant differences were detected in both IPI status (multivariate: *p* = 0.013) and MAX expression (multivariate: *p* = 0.022) (Table 2A). The long-term survival rate associated.

**Table 2:** Univariate and multivariate analyses of overall survival (OS) and progression-free survival (PFS).

Factors	Univariate		Multivariate	
	Hazard ratio (CI)	*P* value	Hazard ratio (CI)	*P* value
(A) OS				
Sex male/female	1.680 (0.56–6.15)	0.367		
IPI IH and H/L and LI	4.304 (1.39–13.7)	0.012	4.434 (1.39–14.7)	0.013
ALK positive/negative	1.111 (0.30–3.33)	0.860		
MYC positive/negative	1.754 (0.52–8.00)	0.383		
MAX negative/positive	4.192 (1.39–12.7)	0.012	3.908 (1.23–13.0)	0.022
(B) PFS				
Sex male/female	1.030 (0.41–2.80)	0.951		
IPI IH and H/L and LI	3.040 (1.07–8.18)	0.037	3.167 (1.09–8.78)	0.034
ALK positive/negative	1.500 (0.52–3.94)	0.437		
MYC positive/negative	1.495 (0.54–4.81)	0.452		
MAX negative/positive	3.200 (1.13–8.49)	0.029	2.874 (0.99–8.01)	0.044

IPI, International Prognostic Index; ALK, Anaplastic lymphoma kinase; MAX, MYC-associated factor X.

Univariate and multivariate analyses for OS also showed a statistically significant difference in both IPI status (univariate: *p* = 0.037, multivariate: *p* = 0.034, respectively) and MAX expression (univariate: *p* = 0.029, multivariate: *p* = 0.044, respectively). Multivariate analysis for OS also showed that both IPI status and MAX expression were independent factors for ALCL (Table 2B). ALK expression was not an independent prognostic factor in this study and there was no statistically difference in PFS and OS between ALK-positive and ALK-negative ALCL. This may be due to similarity of age in these two groups. Although ALK-positive ALCL cases usually have better prognosis than that of ALK-negative ALCL, this difference may be due to the fact that ALK-positive ALCL occurs more frequently at a young patient^17^. These results indicate that decreased MAX expression might be a biomarker of poor prognosis in ALCL.

### Morphological and immunohistochemical features according to MAX expression in ALCL patients

**Figure 4:**
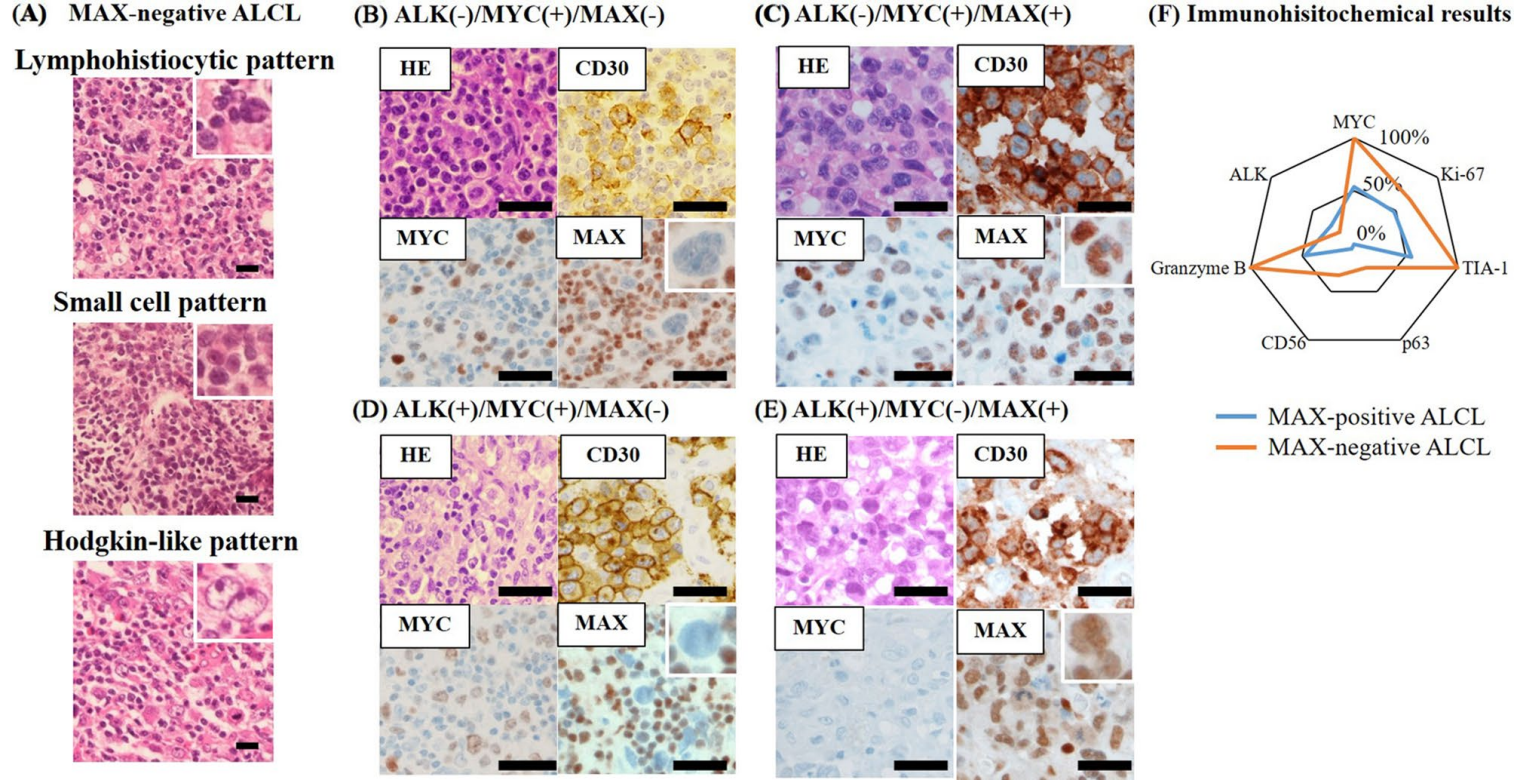
Representative histopathological findings, immunohistochemical findings and results in anaplastic large cell lymphoma (ALCL) cases. (**A**) Representative figures of histological variants, (**B**) representative ALCL case with MYC expression and without Anaplastic lymphoma kinase (ALK) and MYC-associated factor X (MAX) expression, (**C**) representative ALCL case with MYC and MAX expression and without ALK expression, (**D**) representative ALCL case with MYC and ALK expression and without MAX expression, and (**E**) representative ALCL case with ALK and MAX expression and without MYC expression (inset with fourfold magnification), (**F**) the comparison of representative immunohistochemical results between MAX-negative and positive ALCL. Expressions of MYC and cytotoxic molecules were significantly higher in MAX negative-ALCL cases than in MAX-positive ALCL cases. Bars: 50 μm.

To characterize pathological features of MAX-positive and MAX-negative ALCL, we examined morphological patterns of all ALCL patients. ALCL is known to have several morphological patterns. Ninety-two percent of MAX-positive ALCL patients were classified into the so-called common type and only 64% of MAX-negative ALCL patients were classified into this type (*p* = 0.037) (Table 3). The remaining MAX-negative patients exhibited non-common patterns such as lymphohistiocytic pattern, small cell pattern, and Hodgkin-like pattern (Fig. 4A). We also explored the expression of lymphoma-associated markers including CD markers, cytotoxic molecules, and MYC protein. Representative images of immunohistochemical analyses are shown in Fig. 4B–E. Results of immunohistochemical analysis according to MAX expression are shown in Fig. 4F and Supplementary Table S3. There was no significant difference in CD markers between MAX-positive and MAX-negative ALCL. MYC expression was explored in 9 out of 11 MAX-negative ALCL patients and observed in these MAX-negative ALCL patients (9 of 9 patients). The MYC expression of the remaining two cases could not be explored because of insufficient specimen. MYC expression was seen in 54% of MAX-positive ALCL patients (13 of 24 patients) (*p* = 0.007). Expression of cytotoxic molecules TIA-1 and granzyme B was observed in all MAX-negative ALCL patients, but only 55% (12 of 22 patients) and 48% (10 of 21 patients) of MAX-positive ALCL patients, respectively (*p* = 0.013, *p* = 0.017, respectively) (see Supplementary Figure S2A online). Moreover, positive rate of Ki-67, CD56 and p63 known as prognostic marker in ALCL was higher in MAX-negative ALCL than in MAX-positive ALCL (Fig. 4F)^6,18,19^. Granzyme B was expressed in MYC-positive ALCL as well (*p* = 0.010) (see Supplementary Figure S2B online).

**Table 3:** Histological patterns of ALCL according to MAX expression.

Histological pattern	MAX-positive ALCL	MAX-negative ALCL	Total
Common pattern	24 (92%)*	7 (64%)*	31
Other patterns	2 (8%)	4 (36%)	6
Lymphohistiocytic pattern	0	1	1
Small-cell pattern	0	1	1
Hodgkin-like pattern	2	2	4
Total	26 (100%)	11 (100%)	37

MAX, MYC-associated factor X; ALCL, anaplastic large cell lymphoma. *p = 0.037.

### FISH results

We analyzed *TP63* and *DUSP22* rearrangement for ALCL patients without *ALK* rearrangement by FISH. The two ALCL patients with *TP63* rearrangement were included in the MAX-negative ALCL group, whereas all four ALCL patients with *DUSP22* rearrangement were classified into the MAX-positive ALCL group (see Supplementary Table S4A online).

## Discussion

**Figure 5:**
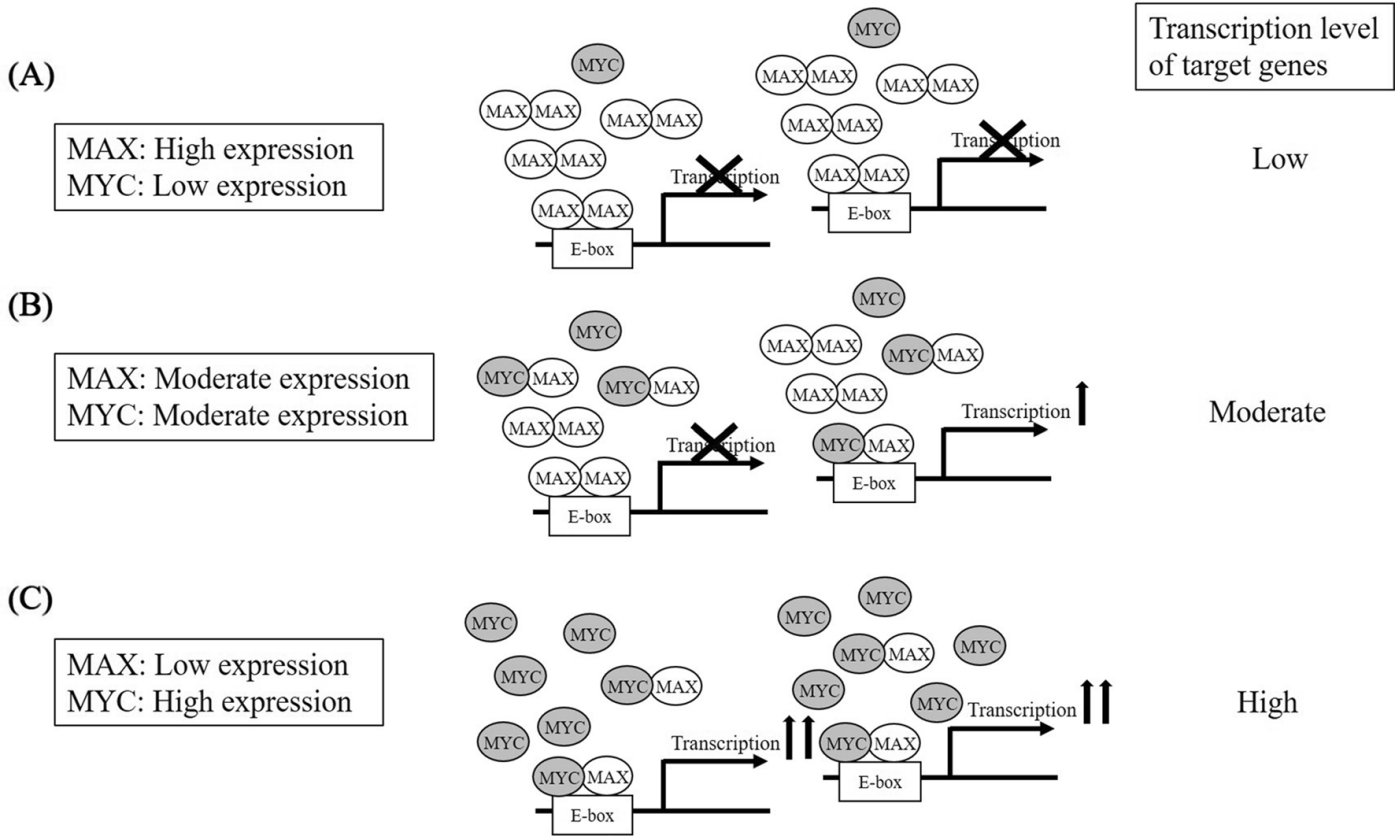
Hypothetical model of interaction between MYC and MAX in ALCL. (**A**) MAX-MAX homodimer can repress the transcription of an MYC-target. (**B**) MYC-MAX heterodimer can activate MYC-target genes, but MAX-MAX homodimer continues to occupy a part of E-boxes. (**C**) MYC-MAX heterodimer can sufficiently activate the transcription of MYC-target genes. ALCL, anaplastic large cell lymphoma.

In this study, we found that decreased MAX expression is a potential adverse prognostic factor in ALCL patients. Our results are comparable to those of a previous report of lymphoblastic lymphoma, in which lack of MAX expression was shown as a worse prognostic factor^16^. MYC translocation or amplification is associated with an aggressive clinical course in ALCL^10–12,20^. As MAX is an essential molecule for the oncogenic activity of MYC to form a heterodimer with MYC protein, it is conceivable that MAX expression affects MYC-driven oncogenic activity in ALCL. Indeed, MYC-positive ALCL patients tended to have a worse prognosis than MYC-negative ALCL patients, although the difference was not statistically significant in our cohort. Moreover, MAX-negative ALCL patients had a worse prognosis than MAX-positive ALCL patients, regardless of MYC expression. This result is rational because MYC transcriptional activity is dependent on MAX. MAX has a biphasic effect on MYC-related transcription activity. Abundant MAX expression generates more MAX-MAX homodimer availability and represses MYC activity through the occupation of DNA binding sites (E-box) of MYC-MAX heterodimer by the homodimer. Decreased MAX protein permits MYC to heterodimerize with MAX instead of MAX-MAX homodimer and to upregulate MYC transcription activity (Fig. 5). In fact, significantly lower MAX expression was observed in ALCL than in PTCL-NOS, while MYC expression levels were similar between groups both in our study and other data. Interestingly, *MAX* mRNA levels in ALCL were lower than those in other mature T-cell lymphomas, regardless of MYC expression. From these results, this peculiar relationship between MYC and MAX as mentioned above may be characteristic for ALCL. Additionally, the detection of MAX expression may aid in the differential diagnosis between ALCL and PTCL-NOS.

From the immunohistochemical results, decreased MAX expression correlated with the expression of cytotoxic molecules such as TIA-1 and granzyme B. Recent reports have shown that expression of cytotoxic molecules may be independent prognostic factors in mature T-cell neoplasms including ALCL^21–23^. Thus, the prognostic difference between MAX-positive and MAX-negative ALCL may be a result of the expression of these molecules. Most of the p63-positve cases and CD56-positive cases were included in MAX-negative group. The high expression of p63, CD56, and high rates of Ki-67 are also known as prognostic markers of ALCL^18,19^. Additionally, several reports recently described that cytotoxic molecules are expressed in ALCL with *TP63* rearrangement but not in ALCL with *DUSP22* rearrangement^4,6^. This finding is in agreement with our results, which showed that the expression of these molecules was detected in 2 ALCLs with TP63 rearrangement (both of them were MAX negative), but not detected in all 4 ALCLs with *DUSP22* rearrangement (all MAX positive) (see Supplementary Table S4B online). These results indicated that *DUSP22* or *TP63* rearrangement might have a partial influence on the expression of cytotoxic molecules through MAX expression in ALCL.

We also tried to characterize morphological features of MAX-positive or MAX-negative ALCL. Our result that MAX-negative ALCL was related to histological features of non-common variants is consistent with the report of Lamant et al., who showed that ALCL with small cell variant or lymphohistiocytic variant had a worse prognosis than ALCL with common variant^24^.

In summary, we demonstrated that (1) decreased MAX expression could be a poor prognostic factor in ALCL, probably through cytotoxic molecules in coordination with MYC, (2) decreased MAX expression is related to histological non-common patterns of ALCL (e.g., patients that had a poor prognosis), and (3) decreased MAX expression might help to distinguish between ALCL and PTCL-NOS.

This study is limited because of a small number of cases so that further extensive studies will be necessary to determine whether the loss of MAX expression is an independent poor prognostic factor in ALCL including the functional analysis of MAX in ALCL.

## Methods

### Cell culture, reagents, and materials

Human anaplastic large cell lymphoma (ALCL) cell lines Karpas-299 (K299) and SUDHL-1; Hodgkin lymphoma (HL) cell lines HDLM2, L540, KMH2, and L428; Burkitt lymphoma cell line Raji; and T lymphoblastic lymphoma cell line Jurkat were maintained in RPMI 1,640 medium (Thermo Fisher Scientific, Waltham**,** MA, USA). Diffuse large B cell lymphoma cell line HT was maintained in Dulbecco's Modified Eagle Medium (DMEM) (Thermo Fisher Scientific). Media were supplemented with 10% (K299, SUDHL-1, HDLM2, L540, KMH2, HT, Raji, and Jurkat) or 20% (L428) fetal bovine serum (Nichirei, Tokyo, Japan), L-glutamine, and antibiotics (penicillin and streptomycin).

### Western blotting

Protein extraction and western blotting were conducted as previously described (1). Briefly, equal amounts of protein (20 μg protein/lane) were separated on a 6–15% sodium dodecyl sulfate gel via polyacrylamide gel electrophoresis and transferred onto polyvinylidene difluoride membranes. Primary antibodies used for western blotting were as follows: anti-MYC-associated factor X (MAX) polyclonal IgG antibody (PoAb) (clone: ab101271, Abcam, Cambridge, UK; 1:2000), anti-MYC monoclonal IgG antibody (MoAb) (clone: Y69, Abcam, 1:5,000), and anti-Actin PoAb (clone: I-19, Santa Cruz Biotechnology, Dallas, TX, USA, 1:1,000).

### RT-PCR and mRNA expression

Total RNA was extracted from cells using TRIzol reagent (Thermo Fisher Scientific) and reverse transcribed using the Super Script™ III First-Strand Synthesis SuperMix (Thermo Fisher Scientific) for PCR according to the manufacturer’s protocol. MAX and GAPDH cDNA sequences were obtained from the National Center for Biotechnology Information GenBank database (https://www.ncbi.nlm.nih.gov/genbank/). cDNAs encoding MAX (NM_002382) and GAPDH (NM_002046) were cloned using PCR from mRNA of lymphoma cell lines. Primers used for PCR were as follows: MAX sense, 5′-AGAGCGACGAAGAGCAACCGA-3′ and MAX anti-sense, 5′-TTGGTCTGCAGTTGGGCA-3′; GAPDH sense, 5′-TGCCTCCTGCACCACCAACT-3′ and GAPDH anti-sense, 5′-CGCCTGCTTCACCACCTTC-3′. PCR was performed with an initial denaturation step at 98 °C for 1 min, followed by 30 cycles of denaturation at 98 °C for 10 s, annealing at 55 °C for 5 s, and extension at 72 °C for 45 s. PCR products were run on a 1% Tris–borate-EDTA (TBE) polyacrylamide gel and stained with Ethidium Bromide EtBr.

### Gene-expression profiling (GEP)

GEP data were obtained from three lymphoma studies (accession numbers GSE19069, GSE58445, and GSE65823) in GEO DataSets^25–27^. Expression and correlation of MYC and MAX mRNA levels in ALCL and other T-cell lymphomas were examined.

### Patients

Biopsy specimens were obtained from 37 patients diagnosed with ALCL and 15 patients diagnosed with PTCL-NOS from 1993 to 2017 at the Department of Pathology of Saitama Medical Center, Saitama Medical University, and Saitama Red Cross Hospital. All patients were diagnosed according to the World Health Organization classification of hematopoietic and lymphoid tissues 2017 (WHO 2017) by four pathologists (J.T., S.M., T.Y., and M.H.) independently, staged according to the Ann Arbor classification, and classified by International Prognostic Index (IPI). We diagnosed ALCL according to the presence of hallmark cells presenting strong and broad CD30 expression to distinguish from a similar entity, PTCL-NOS. Moreover, 15 PTCL-NOS patients strictly diagnosed according to WHO 2017 were included in this study to determine whether MAX expression is a candidate biomarker to differentiate between ALCL and PTCL-NOS. The study was conducted in accordance with the Declaration of Helsinki of 1975, as revised in 2008 and was approved by the ethics committees of Saitama Medical Center, Saitama Medical University, and Saitama Red Cross Hospital. We also obtained informed consent of all cases.

### Immunohistochemical analysis

Immunohistochemical analysis was performed as previously described^28^. Antibodies listed in Supplementary Table S1 were used for immunohistochemical detection. Immunohistochemical staining was performed using Ventana i-View DAB kit reagents (Ventana Medical Systems, Tucson, AZ, USA) and an automated immunostainer (Ventana ULTRA). Protein expression was blindly assessed by two pathologists (T.Y. and J.T.). Immunohistochemical results were defined as positive or negative according to the proportion of positive cells in 5 fields. Criteria used to indicate positive staining were as follows: all CD markers, TIA-1, and granzyme B, > 20% of cancer cells stained^29^; MAX, ≥ 30% of cancer cells stained^16^; MYC, ≥ 40% of cancer cells stained^25^; and Ki-67 and p63, ≥ 70% of cancer cells stained^5,26^. Moreover, the intensity of the MAX-positive signal was scored from 0 to 5 + , and > 3 + was assessed as positive^16^.

### Fluorescent in situ hybridization (FISH)

FISH probes for *TP63* and *DUSP22* were purchased from ZytoVision GmbH (ZytoLight SPEC *IRF4, DUSP22* Dual Color Break Apart Probe, Bremerharven, Germany) and Empire Genomics (*TP63* Break Apart FISH probe, Williamsville, NY, USA), respectively. For *DUSP22*, break apart probe labeled with Spectrum ZyOrange and ZyGreen labeled polynucleotide target sequences mapping to 6p25.3 distal and proximal to the *DUSP22* gene region, respectively. For *TP63*, break apart probe consisted of distal and proximal regions to *TP63* region in 3q28 and were labeled with Spectrum Orange and Spectrum Green, respectively. Images were obtained and analyzed according to routine institutional protocols. Cut-off levels for positive FISH signal were 10% and 4.5% for *DUSP22* and *TP63*, respectively, as previously described^6,25,30^. Total counted numbers of target cells were approximately 100 cells for detection of fracture, and all cases were judged by two or more investigators.

### Statistical analyses

Comparisons between groups for immunohistochemical analysis were carried out using Fisher’s exact test, the Mann–Whitney U test, or the Wilcoxon test. The Kaplan–Meier method and log-rank test were used for comparison of overall survival and progression-free survival between groups separated by immunohistochemical results. Univariate and multivariate Cox regression analyses were performed to test the association between predicted prognostic factors and survival outcome. In all cases, results were considered significant at *p* < 0.05. Statistical testing was performed using JMP12 (SAS, Tokyo, Japan).

## Supplementary information

Supplementary file1 (PDF 942 kb)

## References

[1] Stein H (1985). The expression of the Hodgkin's disease associated antigen Ki-1 in reactive and neoplastic lymphoid tissue: evidence that Reed-Sternberg cells and histiocytic malignancies are derived from activated lymphoid cells. Blood.

[2] Parrilla Castellar ER (2014). ALK-negative anaplastic large cell lymphoma is a genetically heterogeneous disease with widely disparate clinical outcomes. Blood.

[3] Wang X (2017). Expression of p63 protein in anaplastic large cell lymphoma: implications for genetic subtyping. Human Pathol..

[4] King RL (2016). Morphologic features of ALK-negative anaplastic large cell lymphomas with DUSP22 rearrangements. Am. J. Surg. Pathol..

[5] Vasmatzis G (2012). Genome-wide analysis reveals recurrent structural abnormalities of TP63 and other p53-related genes in peripheral T-cell lymphomas. Blood.

[6] Yamashita T (2019). Anaplastic large cell lymphoma with TP63 rearrangement: a dismal prognosis. Pathol. Int..

[7] Cascon A, Robledo M (2012). MAX and MYC: a heritable breakup. Cancer Res..

[8] Manso R (2016). C-MYC is related to GATA3 expression and associated with poor prognosis in nodal peripheral T-cell lymphomas. Haematologica.

[9] Gu Y (2017). Stabilization of the c-Myc protein by CAMKIIγ promotes T cell lymphoma. Cancer Cell.

[10] Moritake H (2011). C-MYC rearrangement may induce an aggressive phenotype in anaplastic lymphoma kinase positive anaplastic large cell lymphoma: identification of a novel fusion gene ALO17/C-MYC. Am. J. Hematol..

[11] Weilemann A (2015). Essential role of IRF4 and MYC signaling for survival of anaplastic large cell lymphoma. Blood.

[12] Inghirami G (1994). Molecular characterization of CD30+ anaplastic large-cell lymphoma: high frequency of c-myc proto-oncogene activation. Blood.

[13] Romero OA (2014). MAX inactivation in small cell lung cancer disrupts MYC-SWI/SNF programs and is synthetic lethal with BRG1. Cancer Discov..

[14] Schaefer IM (2017). MAX inactivation is an early event in GIST development that regulates p16 and cell proliferation. Nat. Commun..

[15] Burnichon N (2012). MAX mutations cause hereditary and sporadic pheochromocytoma and paraganglioma. *Clin. Cancer Res*. Off. J. Am. Assoc. Cancer Res..

[16] Yuza Y, Kawakami M, Takagi K, Yamazaki Y, Urashima M (1999). Max protein expression is associated with survival of children with lymphoblastic lymphoma. Pediatr. Int. Off. J. Jpn. Pediatr. Soc..

[17] Grewal JS (2007). Highly aggressive ALK-positive anaplastic large cell lymphoma with a leukemic phase and multi-organ involvement: a report of three cases and a review of the literature. Ann. Hematol..

[18] Suzuki R (2000). Prognostic significance of CD56 expression for ALK-positive and ALK-negative anaplastic large-cell lymphoma of T/null cell phenotype. Blood.

[19] Wang YF (2012). Clinical and laboratory characteristics of systemic anaplastic large cell lymphoma in Chinese patients. J. Hematol. Oncol..

[20] Raetz EA (2002). The nucleophosmin-anaplastic lymphoma kinase fusion protein induces c-Myc expression in pediatric anaplastic large cell lymphomas. Am. J. Pathol..

[21] Asano N (2007). Cytotoxic molecule expression is predictive of prognosis in Hodgkin's-like anaplastic large cell lymphoma. Histopathology.

[22] Cataldo KA (1999). Detection of t(2;5) in anaplastic large cell lymphoma: comparison of immunohistochemical studies, FISH, and RT-PCR in paraffin-embedded tissue. Am. J. Surg. Pathol..

[23] Jaffe ES, Krenacs L, Raffeld M (2003). Classification of cytotoxic T-cell and natural killer cell lymphomas. Semin. Hematol..

[24] Lamant L (2011). Prognostic impact of morphologic and phenotypic features of childhood ALK-positive anaplastic large-cell lymphoma: results of the ALCL99 study. J. Clin. Oncol. Off. J. Am. Soc. Clin. Oncol..

[25] Crescenzo R (2015). Convergent mutations and kinase fusions lead to oncogenic STAT3 activation in anaplastic large cell lymphoma. Cancer Cell.

[26] Scarfo I (2016). Identification of a new subclass of ALK-negative ALCL expressing aberrant levels of ERBB4 transcripts. Blood.

[27] Iqbal J (2014). Gene expression signatures delineate biological and prognostic subgroups in peripheral T-cell lymphoma. Blood.

[28] Yamashita T, Higashi M, Momose S, Morozumi M, Tamaru JI (2017). Nuclear expression of Y box binding-1 is important for resistance to chemotherapy including gemcitabine in TP53-mutated bladder cancer. Int. J. Oncol..

[29] Broyde A (2009). Role and prognostic significance of the Ki-67 index in non-Hodgkin's lymphoma. Am. J. Hematol..

[30] Wada DA (2011). Specificity of IRF4 translocations for primary cutaneous anaplastic large cell lymphoma: a multicenter study of 204 skin biopsies. Mod. Pathol. Off. J. U. S. Can. Acad. Pathol. Inc..

