# Supplementary material for: Decreased MYC-associated factor X (MAX) expression is a new potential biomarker for adverse prognosis in anaplastic large cell lymphoma
Source: Sci Rep. 2020 Jun 25;10:10391. doi: 10.1038/s41598-020-67500-w (PMC7316730; doi:10.1038/s41598-020-67500-w)

Title: Decreased MYC-associated factor X (MAX) expression is a new potential biomarker for adverse prognosis in anaplastic large cell lymphoma

Author names and affiliations:

Takahisa Yamashita, Morihiro Higashi, Shuji Momose, Akiko Adachi, Toshiki Watanabe, Yuka Tanaka, Michihide Tokuhira, Masahiro Kizaki and Jun-ichi Tamaru

Supplementary figure legends

Supplementary Fig. S1

The reorganized MAX expression compared to CD30 (MAX/CD30 ratio) from two public data (GSE19069 and 6823). MAX/CD30 ratio was significantly lower in ALK+ALCL and ALK-ALCL than PTCL-NOS. ALK+ALCL: ALK-positive anaplastic large cell lymphoma, ALK-ALCL: ALK-negative anaplastic large cell lymphoma, PTCL-NOS: peripheral T-cell lymphoma, not otherwise specified.

Supplementary Fig. S2

Immunohistochemical findings according to MYC-associated factor X (MAX) or MYC expression. (A) Decreased MAX expression correlated with expression of MYC (p=0.007), TIA-1 (p=0.013), and granzyme B (p=0.017). (B) MYC expression correlated with Granzyme B expression (p=0.010) but not TIA-1 expression.

Supplementary Table S1. Antibodies used for immunohistochemical analysis.

Antibody	Clonal	Clone	Company	Dilution
CD2	M	AB75	Leica	1:40
CD3	M	LN10	Leica	1:100
CD4	M	SP35	Roche	Diluted
CD5	M	4C7	Leica	1:20
CD7	M	LP15	Biocare	1:50
CD8	M	C8/144B	Dako	1:100
CD30	M	1G12	Leica	1:40
CD56	M	1B6	Leica	1:50
ALK	M	ALK-1	Dako	1:25
p63	M	4A4	Nichirei	Diluted
TIA-1	M	2G9A10F5	Beckman	1:150
Granzyme B	M	GrB-7	Monosan	1:40
Ki-67	M	MIB-1	Dako	1:100
MYC	M	Y69	Abcam	1:200
MAX	P	Ab101271	Abcam	1:1000

M: monoclonal IgG antibody, P: polyclonal IgG antibody.

Leica: Leica Biosystems, Wetzlar, Germany; Roche: Roche Diagnostics K.K., Tokyo, Japan; Biocare: Biocare Medical, Pacheco, CA, USA; Dako: Dako Japan, Tokyo, Japan; Nichirei: Nichirei Biosciences Inc., Tokyo, Japan; Beckman: Beckman Coulter, Brea, CA, USA; Monosan: Monosan, Uden, Netherlands; Abcam: Abcam, Cambridge, UK.

Supplementary Table S2. MYC and MAX expression in ALCL and PTCL-NOS.

Diagnosis	MYC expression	MAX expression
ALCL	67% (22/33)	70% (26/37)
PTCL-NOS	60% (9/15)	100% (15/15)

MAX: MYC-associated factor X, ALCL: anaplastic large cell lymphoma, PTCL-NOS: peripheral T-cell lymphoma not otherwise specified.

Supplementary Table S3. Immunohistochemical results according to MAX expression.

Antigen	MAX-positive ALCL<br>Number of positive cases (%)	MAX-negative ALCL<br>Number of positive cases (%)	*P* value
CD2	12/20 (60%)	4/9 (44%)	0.688
CD3	10/23 (43%)	3/10 (30%)	0.700
CD4	13/20 (65%)	6/9 (67%)	1.000
CD5	5/21 (24%)	4/9 (44%)	0.389
CD7	1/19 (5%)	1/8 (13%)	0.513
CD8	2/20 (10%)	0/8 (0%)	1.000
CD30	26/26 (100%)	11/11 (100%)	0.690
CD56	1/20 (5%)	3/9 (33%)	0.076
ALK	7/25 (28%)	2/11 (18%)	0.690
MYC	13/24 (54%)	9/9 (100%)	0.007
Ki-67	10/21 (48%)	6/9 (67%)	0.440
p63	0/20 (0%)	2/8 (25%)	0.074
TIA-1	12/22 (55%)	11/11 (100%)	0.013
Granzyme B	10/21 (48%)	8/8 (100%)	0.017

ALCL: anaplastic large cell lymphoma.

Supplementary Table S4. Rearrangement types of ALCL according to the expression of MYC-associated factor X (MAX) or cytotoxic molecules.

(A) MAX expression

Rearrangement type	MAX-positive ALCL	MAX-negative ALCL
ALK	7/25 (28%)	2/11 (18%)
TP63	0/20 (0%)	2/8 (25%)
DUSP22	4/19 (21%)	0/7 (0%)

(B) Cytotoxic molecules

Rearrangement type	TIA-1 expression	Granzyme B expression
ALK	5/6 (83%)	4/6 (67%)
TP63	2/2 (100%)	2/2 (100%)
DUSP22	0/4 (0%)	0/4 (0%)

ALCL: anaplastic large cell lymphoma. ALK: Anaplastic lymphoma kinase, DUSP22: Dual Specificity Phosphatase 22

Supplementary Figure 1

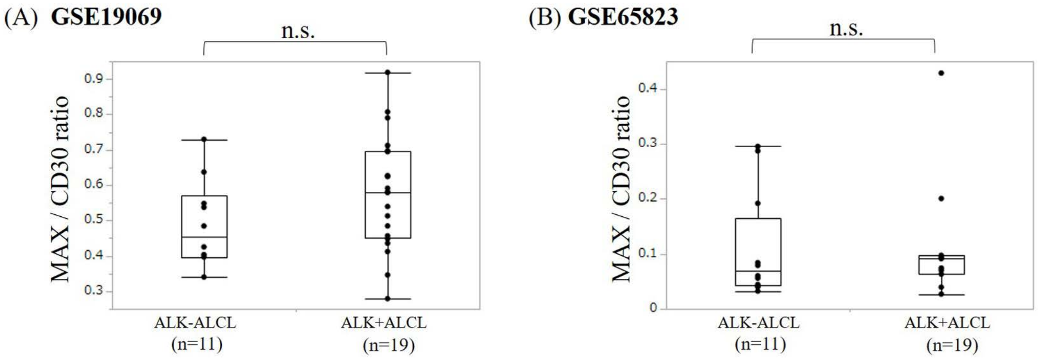

Supplementary Figure 2

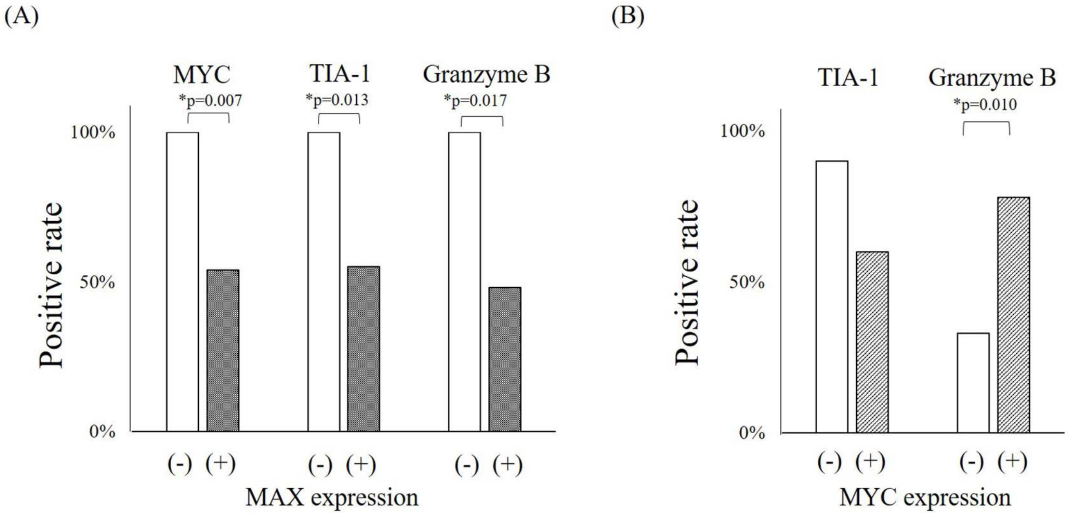

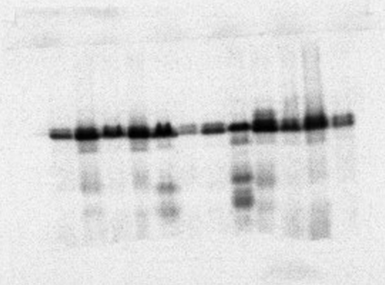

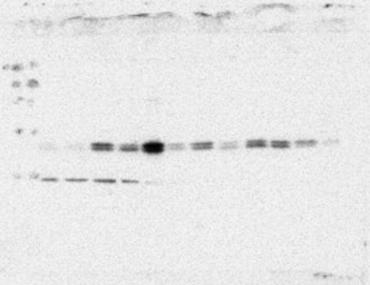

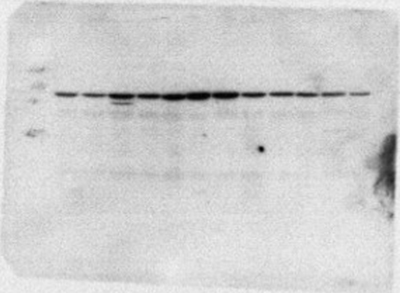

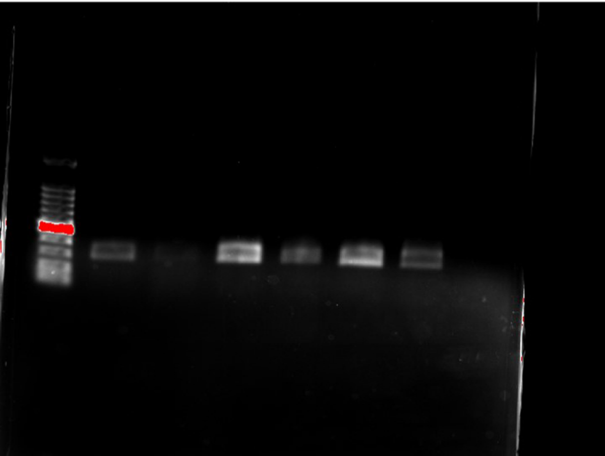

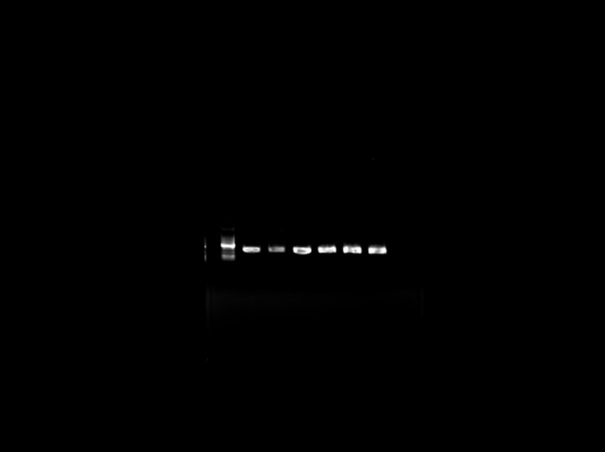

Supplement: Supplementary file 1 — Supplementary file1 (PDF 942 kb) [file 41598_2020_67500_MOESM1_ESM.pdf]
